# Cost Effectiveness of Fibrosis Assessment Prior to Treatment for Chronic Hepatitis C Patients

**DOI:** 10.1371/journal.pone.0026783

**Published:** 2011-12-02

**Authors:** Shan Liu, Michaël Schwarzinger, Fabrice Carrat, Jeremy D. Goldhaber-Fiebert

**Affiliations:** 1 Department of Management Science and Engineering, Stanford University, Stanford, California, United States of America; 2 Equipe ATIP-AVENIR/UMR-S 738 INSERM, Paris Diderot University, Paris, France; 3 UMR-S 707 INSERM, Pierre et Marie Curie University, Paris, France; 4 Department of Medicine, Center for Health Policy and Center for Primary Care and Outcomes Research, Stanford University, Stanford, California, United States of America; Duke University School of Medicine, United States of America

## Abstract

**Background and Aims:**

Chronic hepatitis C (HCV) is a liver disease affecting over 3 million Americans. Liver biopsy is the gold standard for assessing liver fibrosis and is used as a benchmark for initiating treatment, though it is expensive and carries risks of complications. FibroTest is a non-invasive biomarker assay for fibrosis, proposed as a screening alternative to biopsy.

**Methods:**

We assessed the cost-effectiveness of FibroTest and liver biopsy used alone or sequentially for six strategies followed by treatment of eligible U.S. patients: FibroTest only; FibroTest with liver biopsy for ambiguous results; FibroTest followed by biopsy to rule in; or to rule out significant fibrosis; biopsy only (recommended practice); and treatment without screening. We developed a Markov model of chronic HCV that tracks fibrosis progression. Outcomes were expressed as expected lifetime costs (2009 USD), quality-adjusted life-years (QALYs), and incremental cost-effectiveness ratios (ICER).

**Results:**

Treatment of chronic HCV without fibrosis screening is preferred for both men and women. For genotype 1 patients treated with pegylated interferon and ribavirin, the ICERs are $5,400/QALY (men) and $6,300/QALY (women) compared to FibroTest only; the ICERs increase to $27,200/QALY (men) and $30,000/QALY (women) with the addition of telaprevir. For genotypes 2 and 3, treatment is more effective and less costly than all alternatives. In clinical settings where testing is required prior to treatment, FibroTest only is more effective and less costly than liver biopsy. These results are robust to multi-way and probabilistic sensitivity analyses.

**Conclusions:**

Early treatment of chronic HCV is superior to the other fibrosis screening strategies. In clinical settings where testing is required, FibroTest screening is a cost-effective alternative to liver biopsy.

## Introduction

Viral hepatitis C (HCV) is a serious liver disease affecting 180 million people worldwide [1]. In the U.S., 1.3% to 1.9% of the population has been infected with HCV, and 2.7 to 3.9 million people live with chronic infection [2]. Chronic HCV causes liver fibrosis, cirrhosis, and hepatocellular carcinoma (HCC), and is the most common cause of liver transplantation in the US [1].

Current practice guidelines in the U.S. recommend treatment for chronic HCV patients with significant fibrosis progression [1]. For pre-treatment evaluations of patients, liver biopsy is the current gold standard to ascertain liver histology and measure fibrosis progression. However, its expense, risk of side-effects, and potential inaccuracy from sampling and observation errors reduce its utility for frequent liver fibrosis screening [3], [4], [5]. Non-invasive tests of liver fibrosis – including serum markers such as FibroTest (FibroSure) and imaging methods such as FibroScan (transient elastography) – offer potentially viable alternatives [6]. These tests are clinically validated in most common liver diseases caused by hepatitis C, hepatitis B, and alcohol abuse.

Few published studies have addressed the cost-effectiveness of non-invasive tests as alternatives to liver biopsy for determining when to initiate treatment for HCV. A number of studies have investigated test characteristics; some have estimated at a threshold of 0.3, sensitivities and specificities of FibroTest of 74–82% and 57–65% [6], respectively, though this changes with the definition of underlying disease and FibroTest cutoff; others have examined the cost-effectiveness of various treatment options, though generally without considering combinations of screening and treatment. One existing cost-effectiveness analysis of non-invasive screening tests fails to adhere to recommended standards including evaluating options over a lifetime horizon and including quality-of-life considerations [7], [8]. Consequently uncertainties remain about the indications, accuracy, and cost-effectiveness of FibroTest and other non-invasive liver fibrosis screening technologies [3]. Furthermore, recent development in new protease inhibitors to treat HCV, such as telaprevir (Incivek™, Vertex), used in conjunction with pegylated interferon and ribavirin, have significantly improved treatment success rates compared to the standard treatment [9]. The cost-effectiveness of the new treatment is unknown.

We performed a model-based cost-effectiveness analysis of six FibroTest and liver biopsy screening strategies followed by treatment for eligible U.S. chronic HCV patients. We assessed FibroTest's viability as a tool to determine when to initiate treatment by addressing the questions: How should FibroTest be used in the context of chronic HCV, if at all? And how should HCV treatment be offered in combination with periodic screening?

## Materials and Methods

### Model

**Figure 1:**
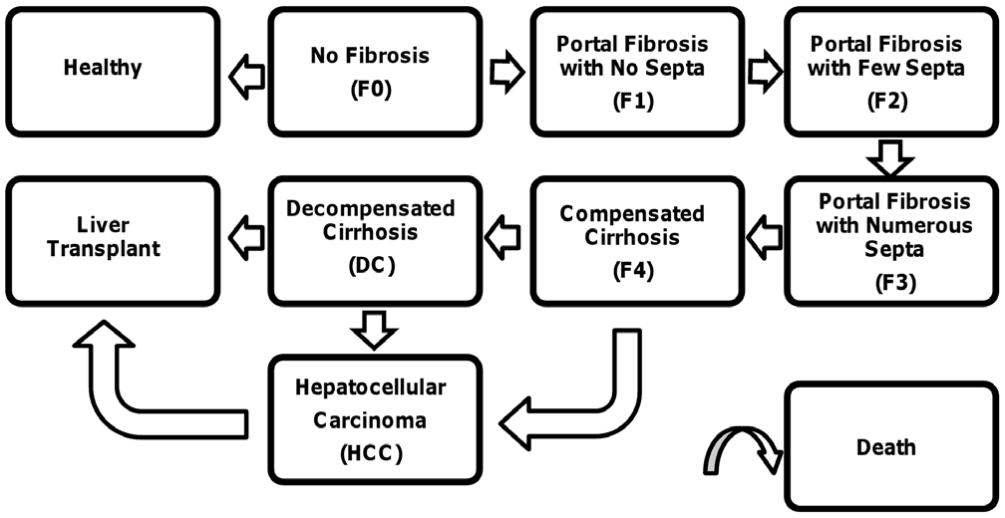
HCV Natural History Model.

The Markov model simulates the lifetime disease progression of a cohort of treatment-naïve men and women who have chronic HCV infections with various stages of liver fibrosis. Progression through fibrosis stages is characterized by the Metavir Scoring system, with possible transitions occurring every 6 months. States include healthy (HCV negative), no fibrosis (F0), portal fibrosis with no septa (F1), portal fibrosis with few septa (F2), numerous septa without cirrhosis (F3), compensated cirrhosis (F4), decompensated cirrhosis (DC), HCC, and liver transplant. Without treatment, complete recovery (returning to the healthy state) is only possible from F0. A proportion of patients who start at F0 are “non-progressors” and do not progress to more severe fibrosis stages. A proportion of patients with decompensated cirrhosis and with HCC receive liver transplants. Death can occur from any state (Figure 1). The model extends a prior, empirically calibrated, model [10]. In the base case, starting age in the model is 40 years old with cohorts age 40 through 70 considered in sensitivity analyses.

**Figure 2:**
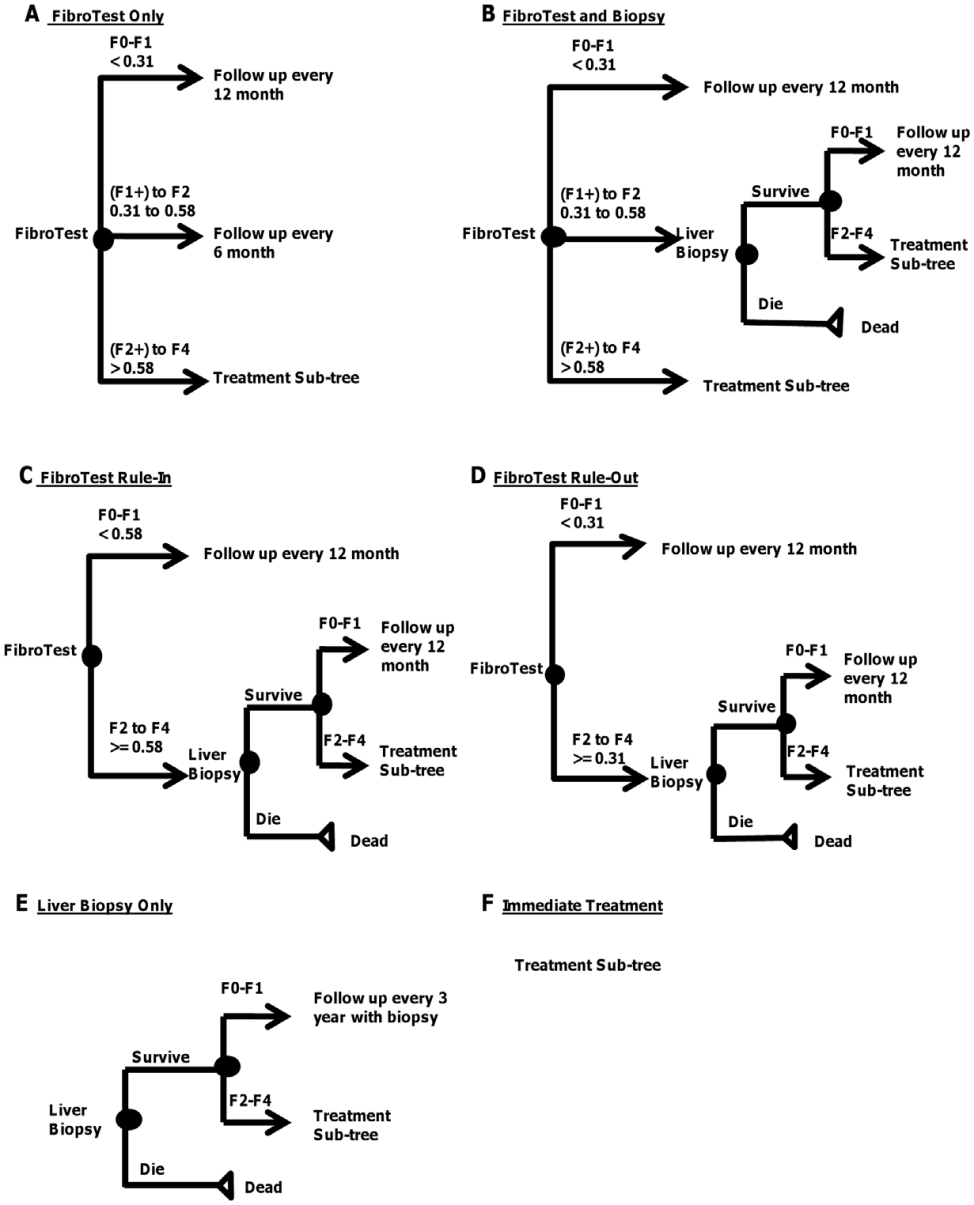
Model Structure; Six Strategies: (A) FibroTest Only; (B) FibroTest and Biopsy; (C) FibroTest Rule-In; (D) FibroTest Rule-Out; (E) Liver Biopsy Only; (F) Immediate Treatment. Note: Panels A–F represent separate clinical strategies that we compare by applying them in our natural history model. “Die” in the figures is to highlight the possibility of death from liver biopsy.

We considered six strategies aimed at detecting fibrosis and beginning treatment to prevent liver disease and death [8], [11]. The strategies considered (Figure 2) are:

#### (A) FibroTest Only

Patients are screened by FibroTest. If the test score is less than 0.31 (mild fibrosis, F0–F1), then repeat FibroTest annually. If the score is between 0.31 and 0.58 (intermediate), then repeat FibroTest every six months. If the test score is greater than 0.58 (significant fibrosis, F2–F4), then begin treatment with no liver biopsy in patients without medical contraindication.

#### (B) FibroTest and Biopsy

Patients are screened by FibroTest. If the test score is less than 0.31, then repeat FibroTest annually with no liver biopsy. If the test score is between 0.31 and 0.58, then follow up with liver biopsy. If liver biopsy indicates significant fibrosis, then begin treatment in patients without medical contraindication. If liver biopsy indicates mild fibrosis, then restart the testing strategy annually. If the test score is greater than 0.58, then begin treatment with no liver biopsy in patients without medical contraindication.

#### (C) FibroTest Rule In

Patients are screened by FibroTest. If the test score is less than 0.58, then repeat FibroTest annually with no liver biopsy. If the test score is greater than or equal to 0.58, then follow up with liver biopsy. If liver biopsy indicates significant fibrosis, then begin treatment in patients without medical contraindication. If liver biopsy indicates mild fibrosis, then restart testing strategy annually.

#### (D) FibroTest Rule Out

Patients are screened by FibroTest. If the test score is less than 0.31, then repeat FibroTest annually with no liver biopsy. If the test score is greater than or equal to 0.31, then follow up with liver biopsy. If liver biopsy indicates significant fibrosis, then begin treatment in patients without medical contraindication. If liver biopsy indicates mild fibrosis, then restart testing strategy annually.

#### (E) Liver Biopsy Only (currently recommended practice)

All patients receive liver biopsy. Those with results showing significant fibrosis without medical contraindication are treated, otherwise they are re-biopsied every 3 years.

#### (F) Immediate Treatment

All patients without medical contraindication are treated without screening for fibrosis.

Do Nothing (HCV natural progression without fibrosis screening or treatment) is only considered in the context of sensitivity analyses.

**Figure 3:**
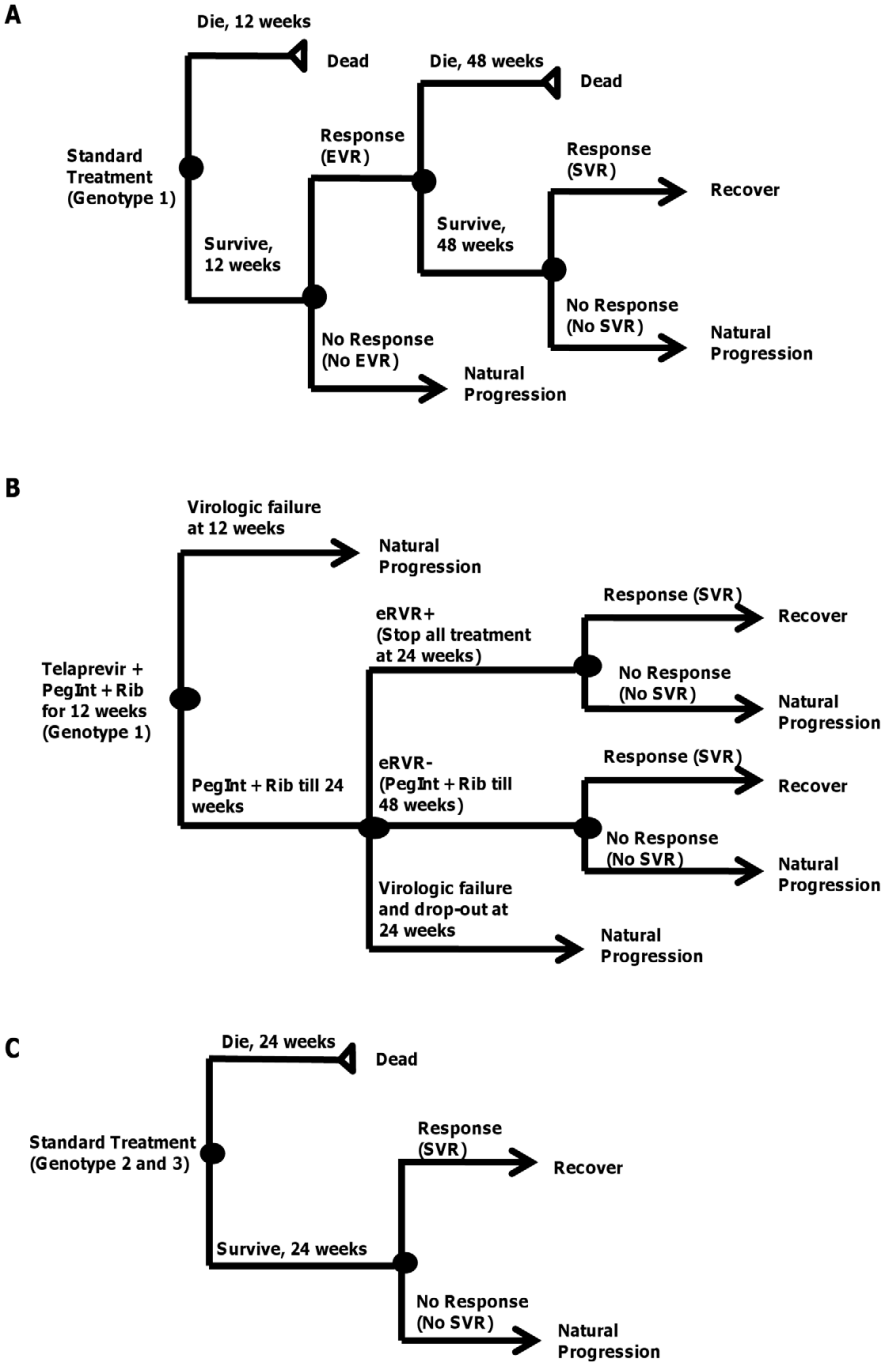
Treatment Sub-tree: (A) Genotype 1 (Standard Treatment); (B) Genotype 1 (Triple Therapy); (C) Genotypes 2 and 3. Note: “Die” in the figures is to highlight the possibility of death from treatment.

Standard treatment includes peginterferon alfa (2a or 2b) and ribavirin for 48 weeks for genotype 1 patients and 24 weeks for patients with genotypes 2 or 3. For genotype 1, an assessment of early viral response (EVR) is modeled at 12 weeks. EVR is defined as a 2 log reduction or complete absence of serum HCV RNA at week 12 of treatment compared with the baseline level. Failure to achieve an EVR is the most accurate predictor of not achieving sustained viral response (SVR) [1]. Non-responders are taken off treatment and resume fibrosis progression. Patients who have undergone complete treatment and achieved SVR transition to a recovered health states stratified by fibrosis severity, and other patients resume fibrosis progression. SVR is defined as the absence of HCV RNA from serum 24 weeks following discontinuation of treatment. (Figure 3 A, C)

We also examined the cost-effectiveness of fibrosis screening in the presence of a new HCV protease inhibitor — telaprevir (Incivek™ Pharmaceuticals) — for treatment naïve genotype 1 patients using response guided therapy in a scenario analysis. Patients receive a 12 weeks course of telaprevir with peginterferon and ribavirin, followed by peginterferon and ribavirin alone for either 12 or 36 weeks depending on extended rapid viral response (eRVR). eRVR is defined as undetectable HCV RNA at week 4 and week 12 (Figure 3 B).

For each strategy, we calculated discounted quality-adjusted life expectancy and total lifetime costs, comparing strategies with incremental cost-effectiveness ratios.

### Data and Sources

We estimated model parameters from extensive review of the published literature and expert opinions.

### Fibrosis

We found wide variations in the literature for the initial distribution of fibrosis stages for chronic HCV patients presented at treatment evaluations. Given the lack of nationally representative data for the US, we derived the prevalence of each fibrosis stage from a large cohort of urban HCV patients (Detroit, Michigan), with 18% F0, 24% F1, 17% F2, 13% F3, 28% F4, and varied the prevalence over a broad range in sensitivity analyses [12].

### Epidemiology

Empirical studies that accurately characterize all phases of HCV natural history and fibrosis progression are lacking due to the asymptomatic acute infection period and long duration (20 to 40 years) between initial infection and progression to end-stage liver disease [1], [10], [13], [14]. Estimates of liver fibrosis progression rates for chronic HCV are heterogeneous [15]. Calibration of a model of HCV to infection prevalence and mortality from liver cancer in the U.S. yields plausible progression rates [16] (see section I in Appendix S1). We incorporated these calibrated rates (stratified by age and gender) in our analysis, and employed the upper and lower ranges in sensitivity analyses (Table 1). Mortality rates from causes other than HCV were derived from 2004 U.S. life tables [17].

**Table 1:** Model Parameter Values: Epidemiology and Cohort Assumptions.*

	Base	Min	Max	Source
Proportion of F0 patients who are non-progressors	0.2420	0.0960	0.7410	[10]
**6 months transition probabilities relating to fibrosis progression**				[10], [16]
Remission (from F0)	0.0060	0.0035	0.0085	
F4 to decompensated cirrhosis (DC)	0.0198	0.0159	0.0247	
Cirrhosis (both F4 and DC) to HCC	0.0104	0.0085	0.0139	
Progression, men by age				[10], [16]
40–49	0.0266	0.0134	0.0464	
50–59	0.0606	0.0358	0.0773	
60–69	0.1046	0.0606	0.1601	
≥70	0.1397	0.0732	0.2126	
Progression, women by age				[10], [16]
40–49	0.0139	0.0065	0.0286	
50–59	0.0320	0.0139	0.0564	
60–69	0.0554	0.0208	0.1113	
70–79	0.0741	0.0397	0.1298	
≥80	0.0997	0.0416	0.1626	
**Liver transplant 6 month probability**				[49]
Liver transplant from DC	0.0253	0	0.2254	
Liver transplant from HCC	0.0780	0.0253	0.2254	
**Disease mortality (6 month rate)**				
Liver transplant mortality	0.0760	0.0719	0.0807	[50]
Post liver transplant mortality	0.0256	0.0250	0.0260	[50]
Decompensated cirrhosis mortality	0.1530	0.0645	0.1975	[10]
HCC mortality	0.2165	0.1595	0.2495	[10]
Liver biopsy mortality (use as probability)	0.0003	0	0.0033	[51]
Treatment mortality (annual rate)	0.0005	0.00025	0.0008	[52]
Cohort starting age^a^	40	40	70	Assumed
Discount rate (annual)	0.03	0	0.05	[7]

*All references included in Table 1–3 are from published literature unless explicitly stated as our assumptions.

a We run the same model with cohorts at different starting age to identify the most cost-effective strategy at each age.

### FibroTest Characteristics

FibroTest is a risk algorithm based on a panel of six blood serum biochemical markers combined with a patient's age and gender that results in a score from 0 to 1 [18] . FibroTest's manufacturer suggests that a score below 0.31 indicates mild fibrosis (F0–F1); 0.32 and 0.58 indicates F1 to F2; and above 0.58 indicates significant fibrosis (F2–F4) [18]. We obtained test characteristics [19] and defined plausible ranges for these test characteristics based on published studies [6], [20], [21], [22], [23], [24], [25], [26], [27]. (Table 2)

**Table 2 pone-0026783-t002:** Model Parameter Values: Screening and Treatment Response Characteristics.

	Base	Min	Max	Source
**Screening Test Characteristics**				
FibroTest (FibroSure)				
Probability for patients with F0–F1				[6], [8], [19], [20]
Test + (>0.58)	0.13	0.06	0.15	
Test − (<0.31), specificity at 0.31	0.68	0.57	0.72	
Probability for patients with F2–F4				
Test + (>0.58), sensitivity at 0.58	0.56	0.35	0.59	
Test − (<0.31)	0.16	0.12	0.29	
Liver biopsy screening frequency (year)	3	3	5	[47]
**Treatment Response Probability**				
Standard treatment (peginterferon and ribavirin)				
Probability(EVR at 12 week), genotype 1	0.71	0.66	0.76	[28]
Probability(SVR | EVR), genotype 1	0.63	0.57	0.69	[28]
Probability(SVR), genotype 2 and 3	0.80	0.60	1.00	[1], [10], [28], [34]
Triple therapy (peginterferon+ribavirin+telaprevir), genotype 1^a^				[9]
Probability(virologic failure at 12 week)	0.03			
Probability(eRVR+, 24 week treatment | non-failure at 12 week)	0.60			
Probability(eRVR−, 48 week treatment | non-failure at 12 week)	0.35			
Probability(SVR|eRVR+, 24 week treatment)	0.89			
Probability(SVR|eRVR−, 48 week treatment)	0.67			
Noncompliance	0	0	0.63	[29]

a The effectiveness listed for triple therapy are for patients with fibrosis stage F0 to F2; for patients with fibrosis stage F3 and F4, SVR is reduced by 20%.

### Treatment Response

A longitudinal study of peginterferon alfa-2b and ribavirin for chronic HCV patients who have undergone EVR assessment at 12 weeks provided the probability of achieving EVR and the probability of SVR for those who achieved EVR [28]. For the new HCV drug telaprevir, we used effectiveness data from the Phase III ADVANCE study [9]. (Table 2)

Patients' initiation of and adherence to treatment can influence the optimal disease management strategy. We modeled full treatment initiation assuming our target population consisted of patients without treatment contraindication. The percentage of eligible patients was varied in sensitivity analysis as research has shown many patients with HCV are not currently treated for reasons including medical and psychiatric co-morbidities, substance abuse, patient refusal or loss to follow-up [29].

### Health Outcomes

Chronic HCV negatively impacts patients' quality of life. To include this important aspect of the disease, we obtained health-state utilities by combining several published studies [10], [30], [31], [32], [33]. There is significant variability among the HCV health-state utility research. We combined estimates to form a consistent set of utilities for all fibrosis stages, HCC, transplant, and post-SVR (see section II in Appendix S1). We modeled utility decrements from biopsy as a one-time disutility of −0.05 (equivalent to a loss of 18 days), standard treatment for one year as −0.11 (equivalent to a loss of 40 days) [30], and assumed −0.165 for one year of triple therapy (equivalent to a loss of 60 days). Decrements were scaled by the actual time on treatment. Because of the variability in estimates, in sensitivity analyses, we widely varied these utilities (see sections II, IV in Appendix S1). (Table 3)

**Table 3:** Model Parameter Values: Quality Weights and Cost.

	Base	Min	Max	Source
**Quality (utilities)** a				[10], [30], [31], [32], [33]
Mild chronic HCV (F0, F1)	0.98	0.70	1.00	
SVR following mild HCV	1.00	0.74	1.00	
Moderate chronic HCV (F2, F3)	0.85	0.66	1.00	
SVR following moderate HCV	0.93	0.71	1.00	
Compensated cirrhosis (F4)	0.79	0.46	1.00	
SVR following F4	0.93	0.60	1.00	
Decompensated cirrhosis	0.72	0.26	0.91	
HCC	0.72	0.15	0.95	
Liver transplant^b^	0.81	0.64	1.00	
Liver biopsy decrement^c^	−0.05	−0.20	0	Assumed
Treatment decrement (standard treatment)^c^	−0.11	−0.20	0	
Treatment decrement (triple therapy)^c^	−0.055	−0.11	0	Assumed
**Cost (2009 USD)**				
Screening test				
Liver biopsy	$1,415	$974	$1,623	[8], [34]
FibroTest (FibroSure)	$236	$100	$295	[8]
Treatment (peginterferon and ribavirin + medical care)				[10], [35], [38], [41]
No EVR, genotype 1 (12 weeks)	$7,383	$5,605	$9,020	
SVR, genotype 1 (48 weeks)	$29,530	$22,420	$36,080	
SVR, genotype 2 and 3 (24 weeks)	$14,765	$11,812	$22,950	
Treatment (telaprevir drug cost for 12 weeks)	$49,200	$36,828	$59,040	[39], [53]
Cost of annual care^d^				[10], [38], [40], [41], [42]
HCV no fibrosis (F0)	$1,610	$150	$2,000	
HCV portal fibrosis (F1, F2)	$1,610	$150	$2,000	
HCV bridging fibrosis (F3)	$1,610	$150	$2,000	
Compensated cirrhosis (F4)	$1,610	$150	$2,000	
Decompensated cirrhosis (DC)	$10,930	$5,470	$16,400	
HCC	$43,510	$21,760	$65,270	
Liver transplant, first year	$143,290	$71,650	$214,930	
Liver transplant, subsequent	$25,020	$12,510	$37,540	

a The quality of life weight for a given age and HCV disease state is computed as the product of the utility associated with the HCV disease state and a mean age-specific quality weight obtained from published data [54], [55].

b Assumed the utility in the post liver transplant state is the same as the utility in F0 state.

c Unlike other utilities these decrement are short-term—only the time period when the intervention occurs.

d Baseline healthcare cost by age is included in the model [56].

### Costs

We included the costs of FibroTest, liver biopsy, treatment, and annual medical care for patients with chronic HCV. FibroTest and liver biopsy costs were obtained from the published literature [8], [34]. Treatment costs include drug cost and medical care cost. To estimate drug costs, we assumed patients received peginterferon alfa-2b 150 mcg once weekly ($584/week, PegIntron™, Schering Corp.; and similarly $580/week, 180 mcg once weekly of peginterferon alfa-2a, Pegasys®, Roche), plus ribavirin 1,000 mg daily ($370.87/week, Rebetol®, Schering Corp.) [35], [36], converting these average wholesale prices to average manufacturer prices using a 0.41 conversion factor [37]. We assumed a medical care cost related to treatment of $10,740 per year based on chronic HCV medical claims data [38]. The cost of telaprevir is reported as $49,200 ($4,100 per week for 12 weeks) for the additional cost of adding telaprevir to standard treatment in a three drug regime [39]. (Table 3)

We estimated the annual care of fibrosis (no treatment) based on medical expenditures in the year following hepatitis C diagnosis [40]. We assumed that patients who obtained SVR post-treatment incurred half of the pre-treatment annual care cost in their associated recovered states [41] and varied this assumption widely in sensitivity analyses [10], [38], [40], [41], [42].

In cost calculations, we adopted a payer perspective, including all direct health care costs, but excluding patient time and transport. We discounted future costs and QALYs by 3% annually. Costs are inflation adjusted using the Consumer Price Index to 2009 [43].

## Results

Among liver fibrosis screening options, strategies using FibroTest are more cost-effective than using Liver Biopsy Only (the current recommended practice) for both men and women with HCV genotype 1, 2, and 3.

**Figure 4:**
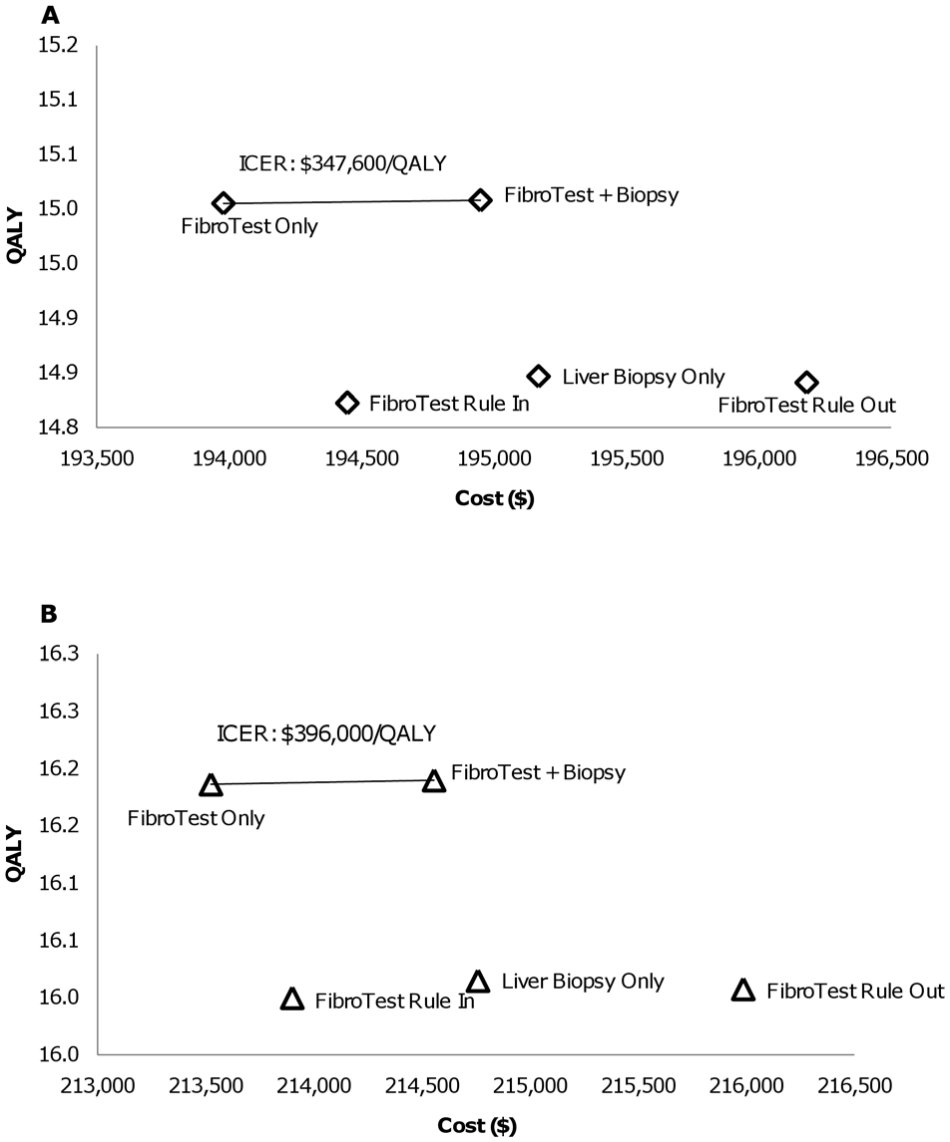
Cost-effectiveness **Results by Gender, Genotype 1 under Standard Treatment (exclude Immediate Treatment): (A) Men; (B) Women.**

**Figure 5:**
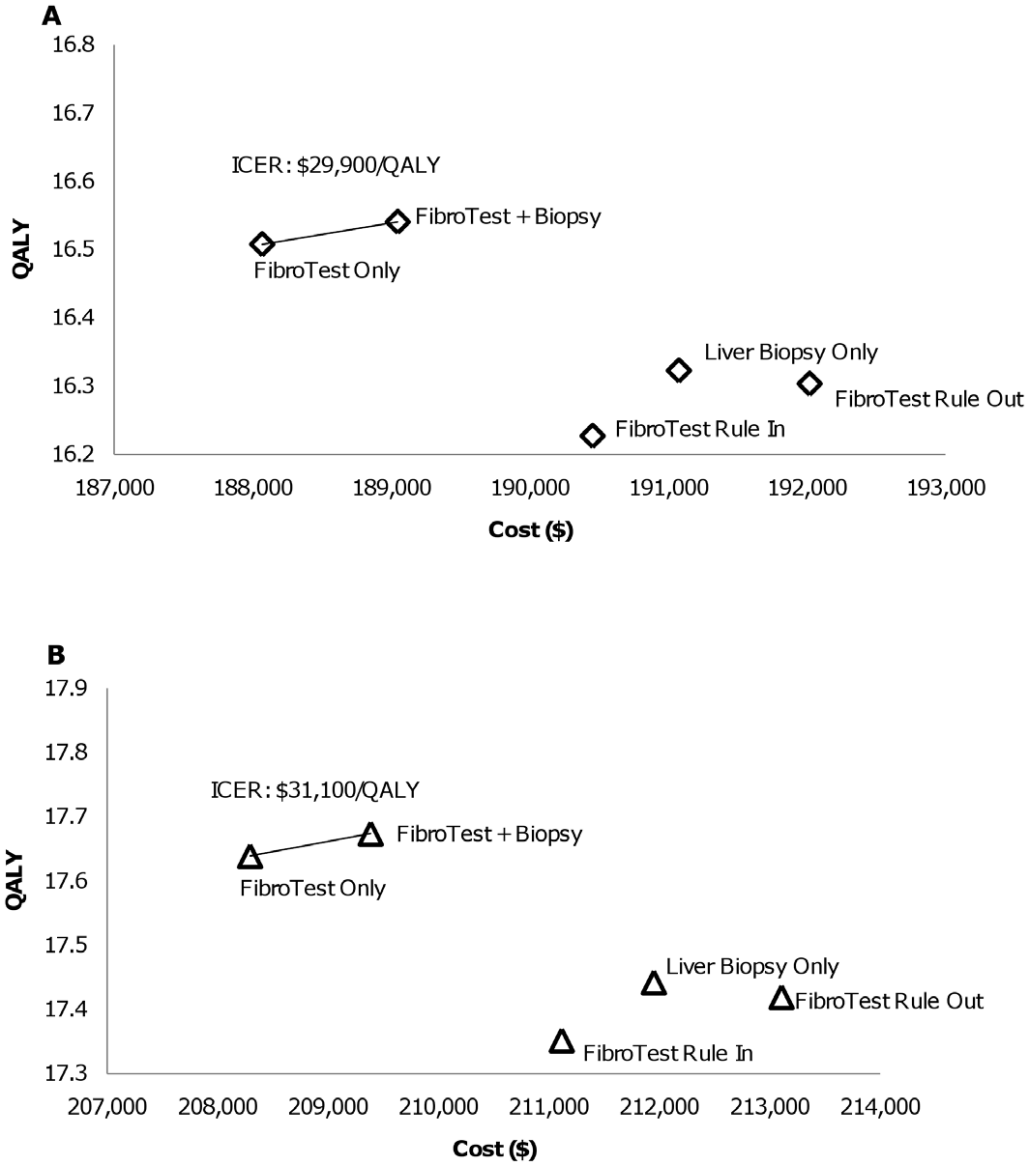
Cost-effectiveness **Results by Gender, Genotype 2 and 3 (exclude Immediate Treatment): (A) Men; (B) Women.** Note: The incremental cost-effectiveness ratio (ICER) is defined as the ratio of the additional costs of an intervention and its additional effects as compared to the next best alternative. i.e. The ICER shown on the figures is between FibroTest Only and FibroTest and Biopsy.

As the current practice in the U.S. is to ascertain that a patient has significant fibrosis progression prior to initiating HCV treatment, especially relevant for genotype 1 patients, we first considered the cost-effectiveness of screening-based strategies only, finding that FibroTest Only costs less and is more effective than Liver Biopsy Only. FibroTest and Biopsy has an ICER of $347,600 compared to FibroTest Only for men and $396,000/QALY for women with genotype 1 (Figure 4), both exceeding thresholds typically used to define cost-effectiveness ($50,000–$100,000/QALY). For patients with genotypes 2 and 3 (Figure 5), FibroTest and Biopsy has an ICER of $29,900/QALY for men and $31,100/QALY for women compared to FibroTest Only. FibroTest and Biopsy is only cost-effective for genotype 2 and 3 patients due to the greater likelihood of their response to treatment. Consequently, the extra liver biopsy and opportunity to initiate treatment based on its results offer more benefits to genotype 2 and 3 patients compare to genotype 1 patients.

If potential management options for chronic HCV included forgoing screening altogether and initiating treatment regardless of fibrosis stage, we find such a strategy cost-effective compared to fibrosis screening (Table 4), with ICERs of $5,400/QALY for men and $6,300/QALY for women with genotype 1 compared to FibroTest Only. All other screening strategies provide less health benefits and cost more. For patients with genotypes 2 or 3, all screening strategies provide less health benefits and cost more.

**Table 4:** Cost-Effectiveness Results by Gender and Genotype, Standard Treatment.

Genotype 1		Cost (US, $)	QALY	ICER ($/QALY)
**Men**	FibroTest Only	193,979	15.01	–
	FibroTest Rule In	194,447	14.82	dominated
	Immediate Treatment	194,514	15.10	5,400
	FibroTest and Biopsy	194,950	15.01	dominated
	Liver Biopsy Only	195,169	14.85	dominated
	FibroTest Rule Out	196,182	14.84	dominated
**Women**	FibroTest Only	213,525	16.19	–
	FibroTest Rule In	213,901	16.00	dominated
	Immediate Treatment	214,101	16.28	6,300
	FibroTest and Biopsy	214,557	16.19	dominated
	Liver Biopsy Only	214,760	16.01	dominated
	FibroTest Rule Out	215,987	16.01	dominated

Genotype 2&3		Cost (US, $)	QALY	ICER ($/QALY)
**Men**	Immediate Treatment	187,547	16.69	–
	FibroTest Only	188,070	16.51	dominated
	FibroTest and Biopsy	189,048	16.54	dominated
	FibroTest Rule In	190,455	16.23	dominated
	Liver Biopsy Only	191,077	16.32	dominated
	FibroTest Rule Out	192,021	16.30	dominated
**Women**	Immediate Treatment	207,829	17.81	–
	FibroTest Only	208,296	17.64	dominated
	FibroTest and Biopsy	209,388	17.67	dominated
	FibroTest Rule In	211,118	17.35	dominated
	Liver Biopsy Only	211,953	17.44	dominated
	FibroTest Rule Out	213,109	17.42	dominated

(ICER: incremental cost-effectiveness ratios. dominated: strategy costs more but achieves less QALY than the previous strategy or a combination of strategies).

The current gold standard, Liver Biopsy Only, provides less health benefit and costs more than strategies using FibroTest or Immediate Treatment across a broad range of assumptions. However, if we consider only screening strategies that include liver biopsy as part of their algorithm, for genotype 1, Liver Biopsy Only is cost-effective compared to FibroTest Rule In (ICER of $29,800/QALY for men and $57,200/QALY for women). For genotypes 2 or 3, Liver Biopsy Only has an ICER below $10,000/QALY compared to FibroTest Rule In.

**Figure 6:**
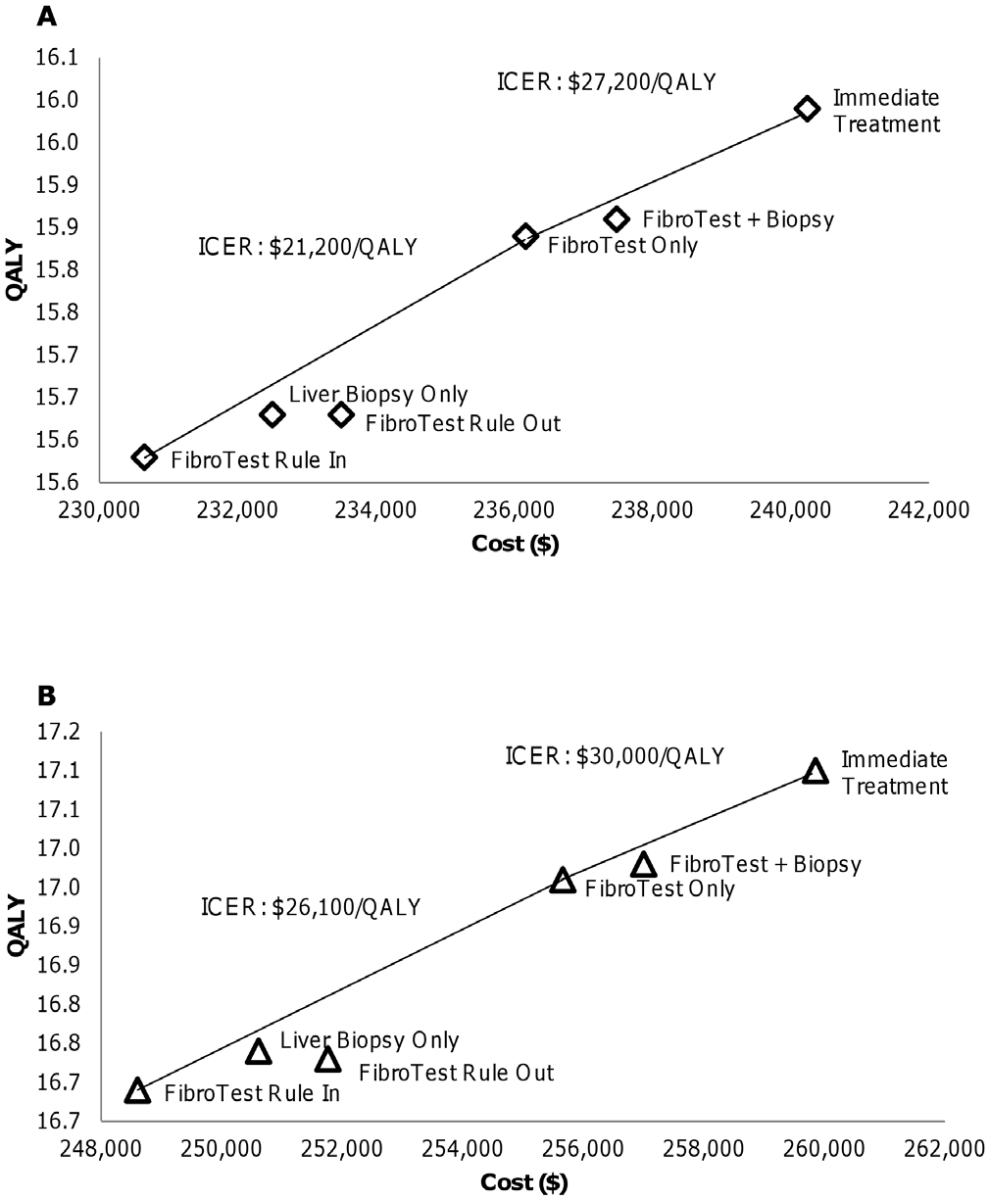
Cost-effectiveness **Results by Gender, Genotype 1 under Triple Therapy with Telaprevir: (A) Men; (B) Women.**

If telaprevir were added to standard treatment in response-guided triple therapy for genotype 1 patients, we find that Immediate Treatment remains cost-effective compared to FibroTest Only based on our assumption of the cost and disutility of telaprevir triple therapy, with an ICER of $27,200/QALY for men and $30,000/QALY for women. (Figure 6) Considering only screening-based strategies but using the new triple therapy, FibroTest Only is cost-effective with an ICER of $21,200/QALY for men and $26,100/QALY for women compared to FibroTest Rule In. (Table 5)

**Table 5:** Cost-Effectiveness Results by Gender and Genotype, Triple Therapy with Telaprevir.

Genotype 1		Cost (US, $)	QALY	ICER ($/QALY)
**Men**	FibroTest Rule In	230,651	15.58	–
	Liver Biopsy Only	232,502	15.63	dominated
	FibroTest Rule Out	233,499	15.63	dominated
	FibroTest Only	236,167	15.84	21,200
	FibroTest and Biopsy	237,482	15.86	dominated
	Immediate Treatment	240,240	15.99	27,200
**Women**	FibroTest Rule In	248,603	16.69	–
	Liver Biopsy Only	250,611	16.74	dominated
	FibroTest Rule Out	251,762	16.73	dominated
	FibroTest Only	255,660	16.96	26,100
	FibroTest and Biopsy	257,002	16.98	dominated
	Immediate Treatment	259,853	17.10	30,000

(ICER: incremental cost-effectiveness ratios. dominated: strategy costs more but achieves less QALY than the previous strategy or a combination of strategies).

Additional base case results can be found in section III in Appendix S1.

### Sensitivity Analyses

Immediate Treatment consistently provided greater health benefit per unit cost compared to the other strategies in one-way sensitivity analyses for all model parameters. In two-way and three-way sensitivity analyses, Immediate Treatment remained the preferred strategy (section IV in Appendix S1). The same conclusion holds for scenario analyses examining patient cohorts aged 50 to 70 years old, increased mortality risks from other causes, slower disease progression rates, improved FibroTest characteristics, reduced SVR for patients with F3 and F4, and a broad range of health utilities estimates (section IV in Appendix S1). For example, while some would argue that older genotype 1 patients should be managed conservatively (i.e., a strategy like “Do Nothing”), we found that for those 70 year-olds with base case fibrosis stage assumption, treatment is still cost-effective though its ICER is higher (Table 6, $31,600/QALY, men).

**Table 6:** Incremental Cost-Effectiveness Ratios ($/QALY) by Cohort Starting Age, Genotype 1 under Standard Treatment, Base Case Fibrosis Stage Distribution.

Men, Age	40	50	60	70
**Do Nothing**	–	–	–	–
**FibroTest Only**	ED	ED	ED	ED
**FibroTest and Biopsy**	D	D	D	D
**FibroTest Rule In**	D	D	D	ED
**FibroTest Rule Out**	D	D	D	D
**Liver Biopsy Only**	D	D	D	D
**Immediate Treatment**	$12,100/QALY	$14,800/QALY	$19,900/QALY	$31,600/QALY

Women, Age	40	50	60	70
**Do Nothing**	–	–	–	–
**FibroTest Only**	ED	ED	ED	ED
**FibroTest and Biopsy**	D	D	D	D
**FibroTest Rule In**	D	D	ED	ED
**FibroTest Rule Out**	D	D	D	D
**Liver Biopsy Only**	D	D	D	D
**Immediate Treatment**	$13,100/QALY	$15,900/QALY	$20,700/QALY	$31,000/QALY

(D: dominated, ED: Extended-Dominated by a combination of Do Nothing and Immediate Treatment).

If treatment was ultimately not given to 100% of eligible patients due to loss to follow-up post screening or medical contraindications discovered post-screening, Immediate Treatment is even more strongly preferred as periodic screening requires resource investment even for those patients who ultimately do not begin treatment.

Immediate Treatment is preferred to screening-based approaches in a probabilistic sensitivity analysis (PSA) (section IV in Appendix S1). Across 10,000 population simulations, at a willingness-to-pay threshold of $50,000/QALY, Immediate Treatment is the preferred strategy more than 99% of the time for both men and women and for all genotypes under standard treatment. For genotype 1 patients under triple therapy using telaprevir, at a willingness-to-pay threshold of $50,000/QALY, Immediate Treatment is the preferred strategy more than 90% of the time for men, and more than 78% of the time for women.

## Discussion

For eligible men and women with chronic HCV of genotype 1, 2, and 3 in the United States, treatment without screening to determine liver fibrosis stage is cost-effective compared to periodic fibrosis screening strategies. Because there may be additional benefits to fibrosis staging prior to treatment (i.e., initiating hepatocellular carcinoma screening for patients with advanced fibrosis) and thus some clinicians may not consider treatment without testing viable, among screening strategies, using FibroTest alone is the next best alternative, and is more effective and less costly than fibrosis screening with liver biopsies. Compared to FibroTest alone, using FibroTest with biopsy reserved for patients with intermediate results has an ICER above $100,000/QALY for genotype 1 and below $50,000/QALY for other HCV genotypes. These finding are robust to multiple assumptions and sensitivity analyses.

This study addresses two important questions — whether to use and how to use non-invasive makers of fibrosis instead of liver biopsy to determine a patient's need for treatment, and the optimal timing to initiate treatment. Many clinicians have shown aversion to non-invasive biomarkers due to the tests' low sensitivity and specificity. Some are concerned that biomarkers fail to make accurate distinctions between mild and severe fibrosis and believe that biopsy may inform treatment decisions in these mid-zones. On the other hand, the apparent failure of serologic markers to distinguish between intermediate stages can be the consequence of classification errors from biopsy - several published studies suggest that when biopsy and marker results are discordant, diagnostic failure of biopsy is much more common than diagnostic failure of biomarkers [44]. Decisions to perform biopsy may depend more on physician preference than on the ability of liver biopsy to influence treatment decisions [45], [46], [47]. We acknowledge the on-going debate around the validity of FibroTest versus that of liver biopsy. However, we find that despite the uncertainties associated with FibroTest's test characteristics, FibroTest Only strategy is preferred over liver biopsy across a broad range of sensitivities and specificities because of its advantage in cost, side effect, and frequency of follow-up. Patients afraid of liver biopsy's side effects may be more accepting of non-invasive tests and consequently these tests may also increase adherence to periodic fibrosis assessment if treatment is withheld. Furthermore, treating all patients (F0–F4) is often cost-effective and therefore distinguishing between mild and significant fibrosis may not be not essential.

Our results contribute to the current debate regarding liver biopsy. Many clinicians recognize liver biopsy's disadvantages. In addition to its cost and risk of adverse effects, liver biopsy is subject to sampling errors (biopsy with a length of 25 mm has a misclassification rate of 25%) [48]. Repeating biopsy every 3–5 years may also be unrealistic due to provider variability and patient non-adherence. Despite this, the National Institute of Health (NIH) 2002 Consensus Statement indicates that liver biopsy still provides unique information on fibrosis and histology, and no panel of serologic markers can provide an accurate assessment of intermediate stages of fibrosis [14]. Similarly, the 2009 American Association for the Study of Liver Diseases (AASLD) guideline recommends liver biopsy in making treatment decisions [1]. However, it recognizes the usefulness of non-invasive tests in defining the presence or absence of advanced fibrosis. Both of the guidelines agree that liver biopsy is not necessary in managing genotype 2 or 3 patients, since their treatment success rate is substantially higher than genotype 1 patients. In support of future amendments to these guidelines, we find that even for genotype 1 patients, both immediate treatment and non-invasive screening appear cost-effective compared to liver biopsy. Furthermore, with the anticipated improvement in treatment success rate for genotype 1 patients, guidelines may soon be revised.

Our results suggest that re-examination of the necessity of screening prior to treatment decision may be appropriate. If treatment is generally effective, additional information obtained via screening may not provide sufficient additional value in guiding clinical decisions, since even with fibrosis stage uncertainty, treatment is likely to be sufficiently beneficial [45], [46]. Our research helps to map out this trade-off between fibrosis stage accuracy and treatment success rate. Though no randomized controlled trials proving that HCV antiviral therapy is associated with long-term clinical benefits, there is a broad literature that strongly suggests this relationship. The lack of long-term evidence may be due to the slow progression of the disease and the short history of the new combination therapy. We found immediate treatment to be cost-effective, given the current treatment effectiveness and anticipated improvements in the future [9]. Our results anticipate new anti-HCV drugs such as telaprevir and boceprevir becoming available that may significantly improve SVR for genotype 1 patients. Even with significantly increased drug costs and potentially increased risk of side-effects, our analyses support immediate treatment without fibrosis screening.

Our analyses and conclusions were robust to a variety of assumptions. Importantly, our conclusions were not sensitive to uncertainties regarding the speed of fibrosis progression and proportion of non-progressors in the cohort. As cost-effectiveness is also influenced by health utilities of HCV health states used in the model, our main conclusion remained robust despite uncertainties regarding these estimates. We also note depending on who is the payer, the cost of treatment can be much lower than our current assumptions (i.e. Federal Supply Schedule for government payers) in which case immediate treatment would appear even more favorable.

Previous research examined the economic outcomes of non-invasive testing in the diagnosis of significant liver fibrosis compared with liver biopsy and recommended against non-invasive testing [8]. The conclusion is made with the assumption that “misdiagnosis” leading to early treatment is harmful to health. The assumption is problematic by disregarding all future benefits and cost. By evaluating a one-time use of non-invasive test, the study ignored one major advantage of non-invasive test that enables more frequent monitoring of fibrosis progression than liver biopsy.

Our study has several limitations. The model does not stratify the population by race, and thus the fibrosis progression and treatment response rates are biased towards whites reflecting the participants in the clinical studies of our source data. Because needed information on genotypes other than 1, 2, and 3 was limited, the model only considers clinical scenarios for genotypes 1, 2, and 3, which is appropriate for a U.S. analysis where these types are most common. We did not consider co-infection with HIV and/or hepatitis B. We defined alternative screening strategies by possible combinations of FibroTest and liver biopsy. Our strategy set is not comprehensive, and we note other screening patterns exist. We did not consider other non-invasive markers and imaging methods such as FibroScan to evaluate liver stiffness. However, for non-invasive tests that are conducted at similar intervals, that have comparable test characteristics and that have comparable costs to FibroTest, our conclusion are also applicable. We also found that treatment without screening to determine liver fibrosis stage would be cost-effective compared to periodic screening strategies. This result was robust to a wide range of sensitivities, specificities, and test costs, and should, therefore, hold for many other non-invasive markers.

Depending on who bears the cost of new antiviral drugs, patients may prefer to wait to initiate treatment until there is evidence of significant fibrosis progression. The model did not include possible future advances in treatment in the base case analysis and allow patients to delay treatment for a later date. The analyses also did not include the benefits of fibrosis screening to patients being able to make an informed choice and, therefore, potentially having a stronger commitment to treatment adherence.

HCV is a serious liver disease affecting up to 4 million Americans. While current recommendations favor liver biopsies prior to treatment initiation, we find that, for the hundreds of thousands of Americans with chronic HCV, other strategies are likely more effective and cost-effective. Management of chronic HCV in the U.S. could be improved by a shift towards strategies that initiate immediate treatment without fibrosis screening or else periodic screening with a non-invasive method followed by treatment for those found likely to have significant fibrosis.

## Supporting Information

Appendix S1 Appendix S1 contains appendices with supporting information.
(DOC)
