# Supplementary material for: Cost Effectiveness of Fibrosis Assessment Prior to Treatment for Chronic Hepatitis C Patients
Source: PLoS One. 2011 Dec 2;6(12):e26783. doi: 10.1371/journal.pone.0026783 (PMC3229483; doi:10.1371/journal.pone.0026783)
Supplement: Appendix S1 — contains appendices with supporting information. (DOC) [file pone.0026783.s001.doc]

**SUPPORTING INFORMATION**

**Cost Effectiveness of Fibrosis Assessment Prior to Treatment for Chronic Hepatitis C Patients**

**Authors:**

Shan Liu, S.M.^1^ (shanliu@stanford.edu)

Michaël Schwarzinger, M.D., Ph.D.^2^ (michael.schwarzinger@inserm.fr)

Fabrice Carrat, M.D., Ph.D.^3^ (carrat@u707.jussieu.fr)

Jeremy D. Goldhaber-Fiebert, Ph.D.^4^ (jeremygf@stanford.edu)

1. Department of Management Science and Engineering, Stanford University, Stanford, CA, USA
2. Equipe ATIP-AVENIR / UMR-S 738 INSERM, Paris Diderot University, Paris, France
3. UMR-S 707 INSERM, Pierre et Marie Curie University, Paris, France
4. Center for Health Policy and Center for Primary Care and Outcomes Research, Department of Medicine, Stanford University, Stanford, CA, USA

**SUPPORTING INFORMATION**

1. **Model Cross-Validation**

The HCV natural history model is cross-validated against Salomon et al. model [16]. Appendix Fig. 1 gives an example of the Markov state probabilities (proportion of the population in each state throughout time) for a cohort of men and women starting with fibrosis stage F0 at age 40, followed by natural fibrosis progression through 120 years of age. The 30 years cumulative probability of developing compensated cirrhosis (F4) for men is 32%, and for women is 9% in the model. These numbers are consistent with Salomon et al.’s results of 30% and 9%, respectively. For a F0 cohort starting at age 40, the 30 year cumulative probability of developing decompensated cirrhosis (DC) is 6.13% for men, and 1.4% for women. The 30 years cumulative probability of developing hepatocellular-carcinoma (HCC) is 3.45% for men, and 0.78% for women. These cumulative probabilities of developing clinical complications are consisted with epidemiologic data.

1. **Utility Preference Order**

We converted between utility estimation methods and combined several sources to achieve a consistent set of utilities for all fibrosis stages, HCC, transplant, and post-SVR, presented in Table 1 of the manuscript. The utilities for moderate chronic HCV (F2, F3), F4, DC, and liver transplant directly came from the Time-Trade-Off (TTO) results in Sherman et al. [31] The utility for mild chronic HCV (F0, F1) and SVR following treatment came from Salomon et al. [10] The utility for SVR following moderate HCV came from the Standard Gamble (SG) results in Chong et al. [32], then converted to TTO using the formula in McLernon et al. [33]. The utility of HCC came from Chong et al. [32] and is assumed to be similar to the utility of DC.

Patients’ utilities for chronic HCV states, recovery states, and post-liver transplant state have a preference ranking order in the model. The utility of:

Mild Chronic HCV ≥ Moderate Chronic HCV ≥ F4 ≥ DC, HCC

SVR following mild HCV ≥ Mild Chronic HCV

SVR following moderate HCV ≥ Moderate Chronic HCV

SVR following cirrhosis ≥ F4

Post-liver transplant ≥ DC, HCC

SVR following mild HCV ≥ SVR following moderate HCV ≥ SVR following cirrhosis

We maintained these preference orders in both deterministic and probabilistic sensitivity analyses by implementing the following structure in the model:

***Deterministic sensitivity analyses:***

d_uMildHCV = max(v_uMildHCV;v_uModHCV; v_uF4; v_uDC; v_uHCC)

d_uModHCV= max(v_uModHCV; v_uF4; v_uDC; v_uHCC)

d_uF4= max(v_uF4; v_uDC; v_uHCC)

d_uDC = v_uDC

d_uHCC = v_uHCC

d_uRecoverMild = max(v_uMildHCV;v_uModHCV; v_uF4; v_uDC; v_uHCC; v_uRecoverMild; v_uRecoverMod; v_uRecoverF4; v_uLT)

d_uRecoverMod = max(v_uModHCV; v_uF4; v_uDC; v_uHCC; v_uRecoverMod; v_uRecoverF4; v_uLT)

d_uRecoverF4= max(v_uF4;v_uDC; v_uHCC; v_uRecoverF4)

d_uLT= max(v_uDC; v_uHCC; v_uLT)

***Probabilistic sensitivity analyses:***

p_uMildHCV = max(dist_uMildHCV; dist_uModHCV; dist_uF4; dist_uDC; dist_uHCC)

p_uModHCV = max(dist_uModHCV; dist_uF4; dist_uDC; dist_uHCC)

p_uF4 = max(dist_uF4; dist_uDC; dist_uHCC)

p_uDC = dist_uDC

p_uHCC = dist_uHCC

p_uRecoverMild = max(dist_uMildHCV; dist_uModHCV; dist_uF4; dist_uDC; dist_uHCC; dist_uRecoverMild; dist_uRecoverMod; dist_uRecoverF4;dist_uLT)

p_uRecoverMod = max(dist_uModHCV; dist_uF4; dist_uDC; dist_uHCC; dist_uRecoverMod; dist_uRecoverF4;dist_uLT)

p_uRecoverF4 = max(dist_uF4 ; dist_uDC; dist_uHCC; dist_uRecoverF4)

p_uLT = max(dist_uDC; dist_uHCC; dist_uLT)

1. **Additional Base Case Results**

***Life Expectancy:***

The US Life Table life (2004) gives the life expectancy of 40 years old men as 37.64 years, and woman as 41.87 years. Running our model with only background mortality produced life expectancies consistent with these numbers. For a cohort of patients starting at age 40, the model computed their life expectancy from each starting fibrosis stage (F0, F1, F2, F3, F4) by gender and genotype (Appendix Tab. 1). “Do Nothing” is HCV natural progression without any intervention strategy.

Our results showed that life expectancy decreases for people with more advanced liver fibrosis. All six intervention strategies result in longer life expectancy than doing nothing, and they produce similar life expectancies within each gender and genotype. For the F0 cohort starting at age 40, prolonged survival may not be the main benefit of treatment. However, for cohorts starting with later stages of fibrosis (F2-F4), prolonged survival becomes a major benefit of treatment in the model. We also observed that men and genotype 1 patients have lower life expectancy than women and patients of genotypes other than 1.

The model computed a life expectancy for patients starting with decompensated cirrhosis to be 5 years (men) and 5.1 years (women). The life expectancy for patients starting with HCC is 5.6 years (men) and 5.8 years (women). These results indicated a slightly higher life expectancy for patients with HCC. This is due to HCC patients having a higher likelihood of receiving liver transplants than DC patients.

***Expected Number of Interventions in the Model:***

The model computed the expected number of FibroTest, liver biopsy, and treatment probability for patients stratified by gender in their life time. For a cohort of patients starting with fibrosis stage (F0, F1, F2, F3, F4) at age 40, the results are shown in Appendix Tab. 2.

The results showed FibroTest rule in (FRI) and rule out (FRO) strategies incur the highest number of expected FibroTest. FibroTest only (FO) and FibroTest with biopsy (F+B) strategies generate the highest likelihood of treatment for people with mild fibrosis (F0, F1) compare to all strategies except Immediate Treatment. If all patients started after treatment initiation stage (F2+) in the model, the expected number of test, biopsy and treatment are similar within each strategy for starting fibrosis stage of F2, F3, and F4. Gender difference is minimal in this analysis.

1. **Additional Sensitivity Analysis Results**

***Deterministic sensitivity analysis:***

We performed one-way sensitivity analyses on all model parameters for all gender and genotypes under standard treatment. In all cases, Immediate Treatment remains to be preferred strategy—having the highest Net Monetary Benefit (NMB) out of the six strategies for all gender and genotypes. NMB is defined as “total QALY × willingness-to-pay – total cost.” We used willingness-to-pay (WTP) of $50,000/QALY. Appendix Fig. 2 displays 4 tornado diagrams showing the range of Net Monetary Benefit under Immediate Treatment strategy by varying each parameter from its minimum to maximum values for women with genotype 1.

Two- and three-way sensitivity analyses included different combinations of test characteristics, utility decrement of treatment vs. biopsy, cost of FibroTest vs. biopsy, cost of chronic HCV care vs. recovery cost, and cost of screening test vs. utility decrement of treatment vs. treatment success rate. Immediate Treatment remains to be cost-effective.

***Scenario Analyses:***

In the base case, we assumed that the occurrence of non-hepatic deaths was the same in chronic HCV population as in the general population. We relaxed this assumption to examine the effect of increased risk for premature non-liver deaths in our cohort. We report here a scenario analysis where we increased the background mortality experienced by individuals in our model three times above the general population of the same age and gender. Immediate Treatment remained cost-effective.

We conducted sensitivity analysis on fibrosis progression rates, using all minimum progression rates from Salomon et al. [10] in all age groups and by gender. Immediate Treatment remained the preferred strategy. In addition, we used 50% of Salomon et al.’s lower bound on progression rates in all age groups and by gender. Result showed that the cost-effectiveness frontier included FibroTest Rule In, FibroTest Only, and Immediate Treatment. Immediate Treatment remained cost-effective.

Pertaining to the discussion on liver biopsy being an imperfect “gold standard,” we conducted one extreme sensitivity analysis making FibroTest as a perfect test; thus eliminating any potential bias against FibroTest compared with liver biopsy. The results showed that Immediate Treatment has an ICER around $12,000/QALY compared with FibroTest Only.

Factors known to affect SVR may include cirrhosis since the absence of bridging fibrosis/cirrhosis was significantly associated with SVR. We conducted a one-way sensitivity analysis on SVR rates for F3 and F4 patients, ranging from 50% of the default SVR rate to the full SVR rate. We found no change in the policy conclusion.

HCV disease-state specific utility estimates are major drivers of the ICER result in the model. We modeled three additional scenarios to explore the effect of utility decrements in the mild, moderate, and F4 HCV disease states (Appendix Tab. 3), assuming all other parameters with their base case values. Result showed that Immediate Treatment remained the preferred strategy.

We investigated uncertainties associated with initial fibrosis stage distributions with two extreme scenarios; all patients starting with F0 stage, or all patients starting with F4 fibrosis stage. For a F0 cohort, we included Do Nothing as a baseline comparator since it is now a feasible strategy. Result showed for genotype 1 patients starting at age 40, the ICER from Do Nothing to FibroTest Only is $31,700/QALY, and the ICER from FibroTest Only to Immediate Treatment is $47,200/QALY for men; and for women, the ICER from Do Nothing to FibroTest Only is $48,600/QALY, and the ICER from FibroTest Only to Immediate Treatment is $68,500/QALY. For genotype 2 and 3, Immediate Treatment has an ICER of $5,900/QALY compared with Do Nothing for men, and $6,500/QALY compared with Do Nothing for women. When we increased the cohort starting age, for genotype 1 patients, Immediate Treatment is cost-effective at the $50,000/QALY threshold for men before age 64, and is cost-effective at the $100,000/QALY threshold for women before age 69. After the threshold, Do Nothing gives the highest net monetary benefit. Therefore, for a cohort of F0 patients, Immediate Treatment is generally cost-effective for younger patients. For a cohort with F4, the result showed Immediate Treatment is cost-effective for both men and women, and for all age and genotypes.

***Probabilistic Sensitivity Analysis, Distribution Parameters:***

We selected 55 parameters for probabilistic sensitivity analysis (PSA) to examine full uncertainty of the data. Each parameter was replaced with either a uniform, triangular, or beta distribution that represented the parameter range in literature. Appendix Tab. 4 presents the distributions for the parameters in our probabilistic sensitivity analysis. We used beta distribution for most parameters that are bounded between 0 and 1. Since beta distribution is a continuous probability distributions defined on the interval between 0 and 1, it can be used as the posterior distribution of the model parameter p of a binomial distribution after observing a − 1 independent events with probability p, and b − 1 with probability 1 − p. The mean of beta distribution is a/ (a + b). We calculated a and b for each model parameter by setting the base case value as the mean of the beta distribution, and a + b as the total number of data points from our source data; thus we can calculate a and b and fully parameterize the beta distribution. We used triangular distributions for most parameters that are not bounded between 0 and 1. The most likely value (i.e. peak of the triangle) of the triangular distribution is set to equal the base case value of the parameter; the base of the triangle represents the parameter’s range. We used uniform distributions for parameters with the highest level of uncertainty from the literature.

All parameters are sampled simultaneously and independently to calculate the Net Monetary Benefit (total QALY × willingness-to-pay – total cost) for each strategy at each simulation. Over 10,000 simulations, we obtained an acceptability curve showing the probability that a strategy achieves the highest Net Monetary Benefit for a range of willingness-to-pay thresholds. Over 10,000 simulations, at a willingness-to-pay threshold of $50,000/QALY, Immediate Treatment is the preferred strategy more than 99% of the time for both men and women and for all genotypes under standard treatment. For genotype 1 patients under triple therapy, at a willingness-to-pay threshold of $50,000/QALY, Immediate Treatment is the preferred strategy more than 90% of the time for men, and more than 78% of the time for women.

Appendix Fig. 3 displays the acceptability curves for genotype 1 patients under either standard treatment or triple therapy. Comparing FibroTest Only with Immediate Treatment, under standard treatment, Immediate Treatment costs less and gains higher QALYs than FibroTest Only for 4% (man) or 3% (woman) of the time, otherwise is cost-effective below the $50,000/QALY threshold; under triple therapy, Immediate Treatment is cost-effective below the $50,000/QALY threshold for 95% (man) or 89% (woman) of the time, otherwise the ICER from FibroTest Only to Immediate Treatment is greater than $50,000/QALY.

**Tables in Supporting Information**

**Appendix Tab. 1. Life Expectancy under Six Strategies by Starting Fibrosis State, Gender (40 year-old Men/Women) and Genotype**

**(FO: FibroTest Only, F+B: FibroTest and Biopsy, FRI: FibroTest Rule In, FRO: FibroTest Rule Out, LBO: Liver Biopsy Only, IT: Immediate Treatment)**

| **Genotype 1** | **F0** | **F1** | **F2** | **F3** | **F4** |
| --- | --- | --- | --- | --- | --- |
| **Do Nothing** | 35.44/40.73 | 32.84/38.69 | 30.41/36.08 | 26.26/31.00 | 18.21/19.04 |
| **FO** | 36.42/41.23 | 34.99/40.11 | 33.66/38.68 | 31.37/35.88 | 26.04/28.28 |
| **F+B** | 36.41/41.22 | 34.98/40.10 | 33.66/38.67 | 31.38/35.88 | 26.30/28.58 |
| **FRI** | 36.41/41.22 | 34.98/40.09 | 33.65/38.66 | 31.34/35.85 | 25.86/28.08 |
| **FRO** | 36.38/41.18 | 34.97/40.07 | 33.65/38.66 | 31.38/35.88 | 26.30/28.58 |
| **LBO** | 36.37/41.17 | 34.96/40.06 | 33.65/38.66 | 31.39/35.88 | 26.45/28.75 |
| **IT** | 36.41/41.23 | 34.99/40.11 | 33.66/38.68 | 31.39/35.89 | 26.46/28.76 |
| **Genotype 2 and 3** | **F0** | **F1** | **F2** | **F3** | **F4** |
| **Do Nothing** | 35.44/40.73 | 32.84/38.69 | 30.41/36.08 | 26.26/31.00 | 18.21/19.04 |
| **Other strategies approximately** | 37.18/41.60 | 36.65/41.20 | 36.18/40.70 | 35.34/39.65 | 32.90/36.00 |

**Appendix Tab. 2. Expected number of FibroTest, Biopsy and Treatment under Six Strategies for 40 Year-Olds by Gender (same across genotype), numbers are separated by starting fibrosis stage (F0, F1, F2, F3, F4)**

**(FO: FibroTest Only, F+B: FibroTest and Biopsy, FRI: FibroTest Rule In, FRO: FibroTest Rule Out, LBO: Liver Biopsy Only, IT: Immediate** Treatment)

| **Scenarios (Men)** | **Expected Number of FibroTest** | **Expected Number of Liver Biopsy** | **Treatment Probability** |
| --- | --- | --- | --- |
| **Do Nothing** | 0 | 0 | 0 |
| **FO** | 6.78, 6.06, 1.78, 1.78,1.73 | 0 | 0.93,0.98, 1, 1, 0.97 |
| **F+B** | 6.61, 5.73, 1.19, 1.19,1.18 | 1.26, 1.12, 0.33, 0.33, 0.33 | 0.92, 0.98, 1, 1, 0.99 |
| **FRI** | 21.1, 13.51, 1.78,1.78,1.7 | 3.18, 2.47, 1, 1, 0.95 | 0.57, 0.93, 1, 1, 0.95 |
| **FRO** | 20.75, 12.95, 1.19,1.19,1.18 | 7, 4.72, 1, 1, 0.99 | 0.57, 0.93, 1, 1, 0.99 |
| **LBO** | 0 | 7.59, 5.21, 1, 1, 1 | 0.57, 0.93, 1, 1, 1 |
| **IT** | 0 | 0 | 1 |
| **Scenarios (Women)** | **Expected Number of FibroTest** | **Expected Number of Liver Biopsy** | **Treatment Probability** |
| **Do Nothing** | 0 | 0 | 0 |
| **FO** | 6.99, 6.65, 1.78, 1.78,1.73 | 0 | 0.93, 0.99, 1, 1, 0.97 |
| **F+B** | 6.87, 6.41, 1.19, 1.19,1.18 | 1.31, 1.24, 0.33, 0.33, 0.33 | 0.92, 0.99, 1, 1, 0.99 |
| **FRI** | 25.92, 18.76, 1.78,1.78,1.7 | 3.75, 3.13, 1, 1, 0.95 | 0.5, 0.91, 1, 1, 0.95 |
| **FRO** | 25.61, 18.21, 1.19,1.19,1.18 | 8.51, 6.39, 1, 1, 0.99 | 0.5, 0.91, 1, 1, 0.99 |
| **LBO** | 0 | 9.71, 6.95, 1, 1, 1 | 0.5, 0.90, 1, 1, 1 |
| **IT** | 0 | 0 | 1 |

**Appendix Tab.** 3. Health-State Utility, Base Case and Scenarios Analyses

| **Scenario** | **Base Case** | **One** | **Two** | **Three** |
| --- | --- | --- | --- | --- |
| **Mild Chronic HCV** | 0.98 | 0.85 | 0.85 | 1 |
| **SVR following mild HCV** | 1 | 1 | 1 | 1 |
| **Moderate Chronic HCV** | 0.85 | 0.85 | 0.85 | 1 |
| **SVR following moderate HCV** | 0.93 | 0.93 | 0.93 | 1 |
| **Compensated cirrhosis (F4)** | 0.79 | 0.79 | 0.85 | 1 |
| **SVR following F4** | 0.93 | 0.93 | 0.93 | 1 |
| **Decompensated cirrhosis** | 0.72 | 0.72 | 0.72 | 0.72 |
| **HCC** | 0.72 | 0.72 | 0.72 | 0.72 |
| **Liver transplant** | 0.81 | 0.81 | 0.81 | 0.81 |

**Appendix Tab. 4. Probability Sensitivity Analyses, Distribution Parameters**

|  | **Distribution** | **a** | | **b** | **Min** | **Max** |
| --- | --- | --- | --- | --- | --- | --- |
| **Screening Test Characteristics** |  | |  |  |  |  |
| **FibroTest (FibroSure)** |  | |  |  |  |  |
| **Probability for patients with F0-F1** |  | |  |  |  |  |
| **Test + (>0.58)** | beta | | 141 | 942 | 0.110 | 0.151 |
| **Test - (<0.31), specificity at 0.31** | beta | | 736 | 347 | 0.652 | 0.707 |
| **Probability for patients with F2-F4** |  | |  |  |  |  |
| **Test + (>0.58), sensitivity at 0.58** | beta | | 272 | 215 | 0.514 | 0.602 |
| **Test - (<0.31)** | beta | | 78 | 409 | 0.129 | 0.194 |
| **Epidemiological Parameter** |  | |  |  |  |  |
| **6 months transition probabilities relating to fibrosis progression** |  | |  |  |  |  |
| **Remission (from F0)** | beta | | 20 | 3323 | 0.0037 | 0.0089 |
| **F4 to decompensated cirrhosis** | beta | | 60 | 2970 | 0.0152 | 0.0250 |
| **Cirrhosis (both F4 and DC) to HCC** | beta | | 40 | 3790 | 0.0075 | 0.0139 |
| **Progression, men by age** |  | |  |  |  |  |
| **40-49** | beta | | 9 | 329 | 0.0123 | 0.0462 |
| **50-59** | beta | | 30 | 465 | 0.0413 | 0.0832 |
| **60-69** | beta | | 20 | 171 | 0.0655 | 0.1520 |
| **≥70** | beta | | 15 | 92 | 0.0814 | 0.2117 |
| **Progression, women by age** |  | |  |  |  |  |
| **40-49** | beta | | 10 | 709 | 0.0067 | 0.0237 |
| **50-59** | beta | | 8 | 242 | 0.0139 | 0.0571 |
| **60-69** | beta | | 5 | 85 | 0.0185 | 0.1111 |
| **70-79** | beta | | 10 | 125 | 0.0364 | 0.1237 |
| **≥80** | beta | | 7 | 63 | 0.0418 | 0.1797 |
| **Liver transplant 6 month probability** |  | |  |  |  |  |
| **Liver transplant from DC** | triangular | |  |  | 0 | 0.2254 |
| **Liver transplant from HCC** | triangular | |  |  | 0.0253 | 0.2254 |
| **Proportion of F0 patients who are non-progressor** | beta | | 5 | 16 | 0.0867 | 0.4366 |
| **Mortality (6 month rate)** |  | |  |  |  |  |
| **Liver transplant mortality** | uniform | |  |  | 0.0719 | 0.0807 |
| **Post liver transplant mortality** | uniform | |  |  | 0.0250 | 0.0260 |
| **Decompensated Cirrhosis mortality** | beta | | 20 | 111 | 0.0966 | 0.2188 |
| **HCC mortality** | beta | | 40 | 145 | 0.16 | 0.2782 |
| **Live Biopsy mortality (probability)** | beta | | 30 | 98415 | 0.00021 | 0.00042 |
| **Treatment mortality (annual)** | beta | | 6 | 11235 | 0.0002 | 0.001 |
| **Treatment Response Probability** |  | |  |  |  |  |
| **Standard treatment (peginterferon and ribavirin)** |  | |  |  |  |  |
| **Probability(EVR at 12 wk), genotype 1** | beta | | 228 | 93 | 0.6595 | 0.7585 |
| **Probability(SVR | EVR), genotype 1** | beta | | 144 | 85 | 0.5654 | 0.6901 |
| **Probability (SVR), genotype 2 and 3** | beta | | 358 | 90 | 0.7608 | 0.8349 |
| **Triple therapy (peginterferon+ribavirin+telaprevir), genotype 1** |  | |  |  |  |  |
| **Probability(SVR|eRVR+, 24 week treatment)** | triangular | |  |  | 0.71 | 0.98 |
| **Probability(SVR|eRVR-, 48 week treatment)** | triangular | |  |  | 0.54 | 0.74 |
| **Quality (utilities)** |  | |  |  |  |  |
| **Mild Chronic HCV (F0, F1)** | beta | | 5.88 | 0.12 | 0.8166 | 1 |
| **SVR following mild HCV** | beta | | 5.88 | 0.12 | 0.8166 | 1 |
| **Moderate Chronic HCV (F2, F3)** | beta | | 38 | 7 | 0.7265 | 0.9336 |
| **SVR following moderate HCV** | beta | | 34 | 2 | 0.8508 | 0.9930 |
| **Compensated cirrhosis (F4)** | triangular | |  |  | 0.6 | 0.8 |
| **SVR following F4** | beta | | 34 | 2 | 0.8508 | 0.9930 |
| **Decompensated cirrhosis (DC)** | triangular | |  |  | 0.6 | 0.8 |
| **HCC** | triangular | |  |  | 0.6 | 0.8 |
| **Liver transplant** | beta | | 8 | 2 | 0.5175 | 0.9719 |
| **Liver Biopsy decrement** | triangular | |  |  | -0.2 | 0 |
| **Treatment decrement (standard treatment)** | triangular | |  |  | -0.2 | 0 |
| **Treatment decrement (triple therapy)** | triangular | |  |  | -0.11 | 0 |
| **Cost (2009 USD)** |  | |  |  |  |  |
| **Screening test** |  | |  |  |  |  |
| **Liver Biopsy** | triangular | |  |  | $974 | $1,623 |
| **FibroTest (FibroSure)** | triangular | |  |  | $100 | $295 |
| **Treatment (Peg Interferon and ribavirin, medical care)** |  | |  |  |  |  |
| **Genotype 1** | triangular | |  |  | $22,420 | $36,080 |
| **Genotype 2 and 3** | triangular | |  |  | $11,812 | $22,950 |
| **Treatment (telaprevir drug cost for 12 weeks)** | triangular | |  |  | $36,828 | $59,040 |
| **Cost of Annual Care** |  | |  |  |  |  |
| **HCV no fibrosis (F0)** | triangular | |  |  | $150 | $2,000 |
| **HCV portal fibrosis (F1, F2)** | triangular | |  |  | $150 | $2,000 |
| **HCV bridging fibrosis (F3)** | triangular | |  |  | $150 | $2,000 |
| **Compensated cirrhosis (F4)** | triangular | |  |  | $150 | $2,000 |
| **Decompensated. Cirrhosis (DC)** | triangular | |  |  | $5,470 | $16,400 |
| **HCC** | triangular | |  |  | $21,760 | $65,270 |
| **Liver transplant, first year** | triangular | |  |  | $71,650 | $214,930 |
| **Liver transplant, subsequent** | triangular | |  |  | $12,510 | $37,540 |

**Figures in Supporting Information**

**Appendix Fig. 1. HCV Natural History: Markov State Probability Analysis, (A) Men; (B) Women**

1. **Men**

1. **Women**

**Appendix Fig. 2. One-way Sensitivity Analysis, Tornado Diagram (Genotype 1, Woman) showing the range of Net Monetary Benefit under Immediate Treatment: (A) All Cost: costs of chronic HCV care and recovery states, and treatment cost have large effects; (B) All Utilities: utilities of F4, HCC, and moderate HCV have large effects; (C) All Mortalities: mortality of decompensated cirrhosis and HCC have large effects; (D) All Probabilities: treatment response rate and probabilities of progressing to decompensated cirrhosis and HCC have large effects**


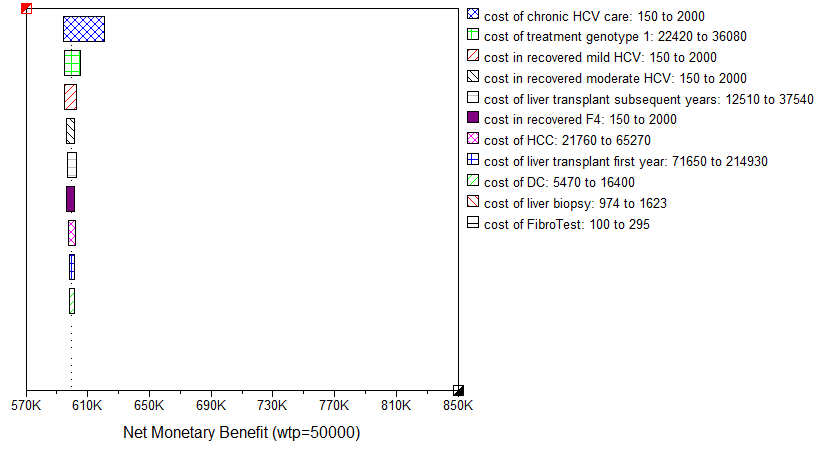


**A.**


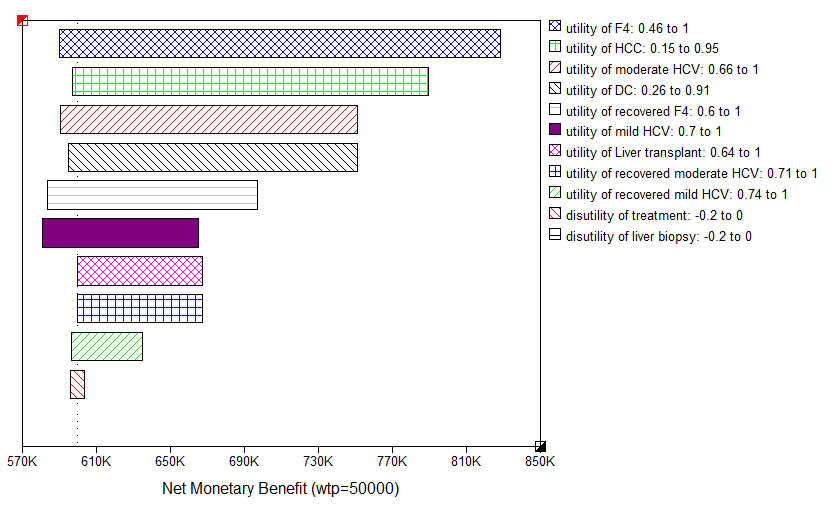


**B.**


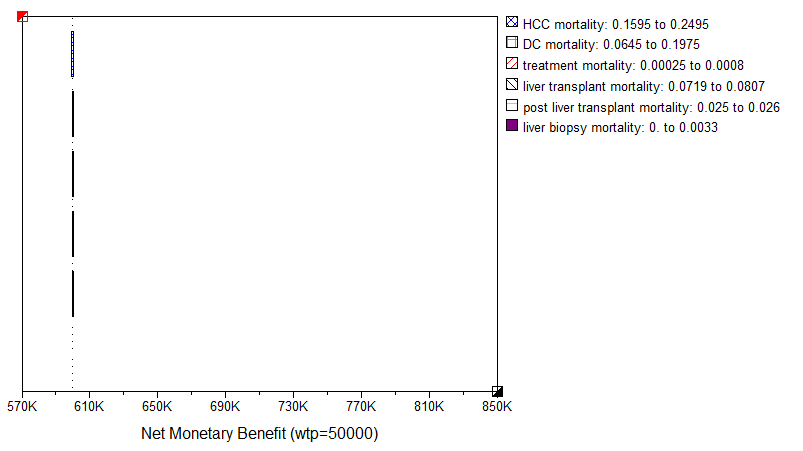


**C.**


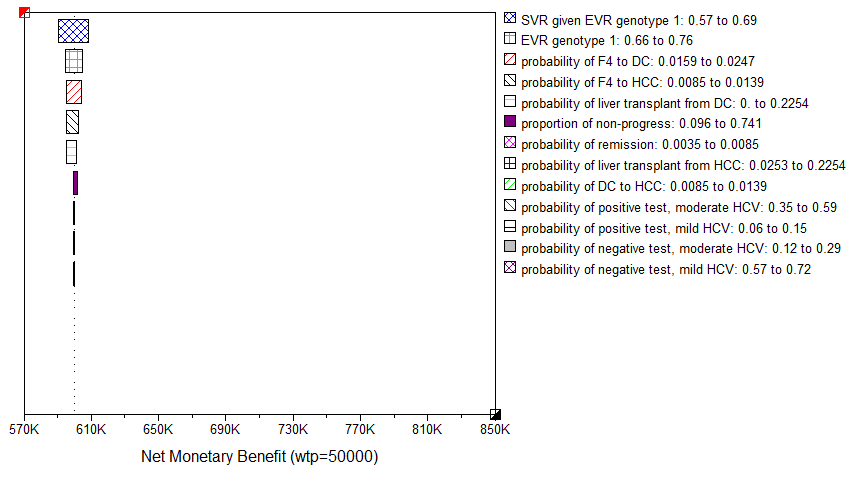


**D.**

**Appendix Fig. 3. Acceptability Curve Genotype 1, 10,000 Samples, (A) Men, standard treatment; (B) Women, standard treatment; (C) Men, triple therapy; (D) Women, triple therapy**

**Note: Strategies whose lines do not appear on the graph were never cost-effective in the probabilistic sensitivity analyses.**

1. **Men, standard treatment**

1. **Women, , standard treatment**

1. **Men, triple therapy**

1. **Women, triple therapy**
